# Preface

**DOI:** 10.3205/cto000127

**Published:** 2015-12-22

**Authors:** Werner Hosemann

**Affiliations:** 1 Department of Otorhinolaryngology, Head and Neck Surgery University Greifswald, Greifswald, Germany

## Preface

Dear Colleagues,

it is a real pleasure for me to present the current issue of the journal including 10 comprehensive scientific reviews and also a special position paper which were presented at the 86^th^ Annual Meeting of the German Society of Oto-Rhino-Laryngology, Head & Neck Surgery, that took place in Berlin in May 2015.

Seven reviews deal with the current status of all relevant clinical aspects of diseases of the paranasal sinuses and the adjacent skull base based on the recent literature. This systematic assessment of the literature aims at summarizing a period of nearly 20 years – since our society chose surgery of the paranasal sinuses as main topic for the last time in 1996. The review on endoscopic endonasal sinus surgery focuses on numerous changes regarding surgical concepts and technical standards. Knowledge and skills of all active sinus surgeons have to keep up with these refinements and improvements. An according paper on the pathophysiology of chronic rhinosinusitis and its conservative therapeutic options is presented. A similar approach is given by the paper on particular, rare rhinologic diseases. Furthermore, there is a paper on the epidemiology of chronic rhinosinusitis confirming that sinusitis has the potential of becoming a widespread disease which is relevant for general healthcare and thus requires special attention. On the other hand, a delineated, however challenging field of “rhino-neurosurgery” has been established by cooperation of rhinologists and neurosurgeons in certain interdisciplinary centers, which will be described in another article. The traumatology of the midface is another field of generally historical character; in this issue the current state-of-the-art will be discussed. Both mentioned interdisciplinary fields have to be promoted in the sense that ENT surgery preserves and secures its position and claim in the practice and applied clinical-practical research. The same is true for relatively new transorbital surgical approaches that are depicted in another paper. 

The increased requirements and demands regarding the treatment of our patients suffering from paranasal sinus diseases can only be fulfilled based on best rational diagnostics and observation of increasing standards of general practice. Both aspects are discussed in the articles on optimized imaging and hygiene of outpatient ENT specific endoscopy. The increasing part of patients with basic diseases outside our discipline whose comorbidities may have a relevant impact on the success of surgical therapy are dealt with in the paper on the management of hemostasis. 

A novelty of this traditional “Congress Issue” is that the presented articles on diseases of the paranasal sinuses and the skull base are completed by a position paper dealing with another topic (i.e. the status of diagnostics and evaluation of swallowing disorders). This position paper originates from a current controversial discussion of healthcare economics. It presents the official point of view of our discipline and delineates the original action framework in an interdisciplinary and healthcare political context.

I want to thank all authors of the papers and the authors of the position paper and I am sure that the present issue will serve as useful source of information and knowledge and will be a reliable argumentative reference for medical offices and hospitals.

Sincerely yours

Prof. Dr. med. Werner Hosemann

President of the German Society of Oto-Rhino-Laryngology, Head & Neck Surgery

(Figure 1 (Fig. 1))

## Figures and Tables

**Figure 1:**
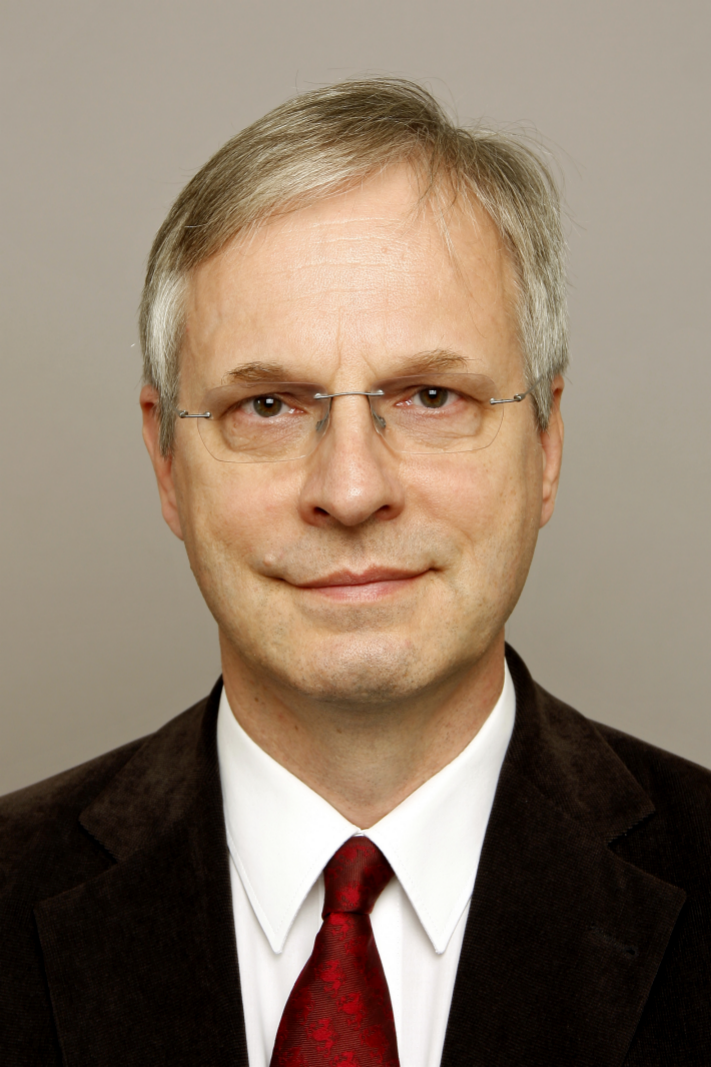
Prof. Dr. med. Werner Hosemann

